# *ANKRD2* Knockdown as a Therapeutic Strategy in Osteosarcoma: Effects on Proliferation and Drug Response in U2OS and HOS Cells

**DOI:** 10.3390/ijms26041736

**Published:** 2025-02-18

**Authors:** Vittoria Cenni, Alberto Bavelloni, Cristina Capanni, Elisabetta Mattioli, Federico Bortolozzo, Snezana Kojic, Giulia Orlandi, Jessika Bertacchini, William L. Blalock

**Affiliations:** 1 Institute of Molecular Genetics “Luigi Luca Cavalli-Sforza”, National Research Council (IGM-CNR), Unit of Bologna, 40136 Bologna, Italy; ccapanni@area.bo.cnr.it (C.C.); elisabetta.mattioli@cnr.it (E.M.); federicobortolozzo@cnr.it (F.B.); jessika.bertacchini@unimore.it (J.B.); william.blalock@cnr.it (W.L.B.); 2 IRCCS Istituto Ortopedico Rizzoli, 40136 Bologna, Italy; 3 Laboratory of Experimental Oncology, IRCCS Istituto Ortopedico Rizzoli, 40136 Bologna, Italy; alberto.bavelloni@ior.it; 4 Group for Muscle Cellular and Molecular Biology, Institute of Molecular Genetics and Genetic Engineering, University of Belgrade, 11042 Belgrade, Serbia; snezana.kojic@imgge.bg.ac.rs; 5 Department of Surgery, Medicine, Dentistry and Morphological Sciences with Interest in Transplant, Oncology and Regenerative Medicine, University of Modena and Reggio Emilia, 41124 Modena, Italy; giulia.orlandi90@unimore.it

**Keywords:** Ankrd2, osteosarcoma, cancer progression, chemotherapy sensitivity, targeted therapy, nuclear lamina

## Abstract

Ankrd2, a mechanoresponsive protein primarily studied in muscle physiology, is emerging as a player in cancer progression. This study investigates the functional role of Ankrd2 in osteosarcoma cells, revealing its critical involvement in cell proliferation and response to chemotherapeutic drugs. We showed that Ankrd2 knockdown impairs the activation of PI3K/Akt and ERK1/2 pathways, reduces levels of cell cycle regulators including cyclin D1 and cyclin B, and counteracts the expression of nuclear lamin A and lamin B, disrupting nuclear morphology and DNA integrity. Strikingly, the loss of Ankrd2 enhances the sensitivity of osteosarcoma cells to doxorubicin and cisplatin, highlighting Ankrd2 potential as a therapeutic target to improve chemotherapeutic efficacy. Defining a novel mechanistic role for Ankrd2 in promoting tumor progression, we propose that Ankrd2 reduction could be exploited as an adjuvant strategy to enhance the efficacy of chemotherapy, offering new therapeutic opportunities for OS treatment.

## 1. Introduction

Osteosarcoma (OS) is a disease that primarily afflicts children, adolescents, and young adults [1]. OS, Ewing sarcoma, and chondrosarcoma represent 90–95% of all primary bone sarcomas, which account for approximately 0.2% of all malignant tumors (SEER Cancer Statistics Factsheets: https://seer.cancer.gov/statfacts/html/bones.html, accessed on 22 April 2019). The early diagnosis of OS results in a 70% overall survival rate [2]. Being a silently progressing cancer [3], estimates suggest that about 30% of patients with OS already have detectable metastases at the time of initial diagnosis; however, of those individuals that initially present without any detectable metastases, approximately 90% will develop metastatic disease in 6 months to 3 years. While the primary tumor can be controlled with chemotherapy and surgery, it is the associated metastases that are lethal. Metastatic OS is most often non-operable and poorly responsive to most current therapies [3]. Clinical treatment of OS consists of the surgical ablation of the tumor, supported by chemotherapy and/or radiotherapy [4]. Chemotherapy basically includes presurgical treatment with a combination of high-dose of methotrexate, adriamycin (the commercial name for doxorubicin), and platinol (the commercial name for cisplatin), also known as MAP, with additional cycles following post-surgical recovery from surgery [5]. Because of the severe side effects of MAP treatment, great efforts are currently aimed at finding viable alternatives to their use.

The ANKyrin Repeat Domain-containing protein 2, Ankrd2, is a mechanoresponsive protein, mainly known for its function in regulating the differentiation and the mechanoresponse of muscle fibers [6]. In differentiated cells, Ankrd2 interacts with sarcomeric proteins including alpha actinin and titin [7,8]. Upon sensing mechanical strain, Ankrd2 shuttles to the nucleus where it is supposed to modulate the transcription of genes involved in the adaptive response [6]. Ankrd2 interacts indeed with several nuclear proteins, including PML, YB1 (a DNA/RNA-binding protein implicated in the regulation of transcription and translation), and BRD3, which binds hyperacetylated chromatin [8,9]. Nonetheless, Ankrd2 also interacts with proteins involved in cell cycle, including p53 [8] and Akt [10], or with proteins with a role in the modulation of their activity, such as the atypical MAPK6 [11]. Our previous studies identified Ankrd2 as a novel substrate of Akt2 [10]. In muscle cells, indeed in response to oxidative stress, Ankrd2 is phosphorylated by Akt2 and translocates to the nucleus [10], where supporting the activity of transcription factors, nkrd2 modulates the expression of proteins involved in cellular proliferation and differentiation [10,12]. Our findings further demonstrated that, in muscle cells, upon exposure to free radicals or reactive oxygen species (ROS), Ankrd2 interacts with lamin A and lamin C, two critical components of the nuclear lamina [13]. Binding to nuclear lamins may assist Ankrd2 bidirectional shuttling to and from the nuclei. Intriguingly, we also demonstrated that the pathogenic mutants of lamin A/C, causative of muscular dystrophies, triggered a basal nuclear recruitment of Ankrd2 in the absence of ROS, suggesting the existence of a significant interplay between these proteins [13]. 

Although specific roles for Ankrd2 in bone physiology have not been described yet, based on its involvement in cell proliferation and mechanotransduction, as well as on its interaction with oncogenes, we hypothesized that Ankrd2 may play a role in osteosarcoma. A study performed by us on OS-derived cell lines demonstrated that the reduction in Ankrd2 expression had an inhibitory effect on their proliferation [14]. Suggesting that Ankrd2 might have a role in supporting cancer progression, our findings demonstrated that it was not the overexpression rather the absence of Ankrd2 that had remarkable effects on the pathophysiology of OS cells [14].

In the present study, to delve deeper into the functional and mechanistic roles of Ankrd2 in OS progression, we explored the consequences of its absence at the molecular level. By exploiting a combinational approach consisting of reverse-phase protein arrays (RPPAs) followed by biochemical validation, we were able to assess that, upon cellular exposure to mitogenic stimuli, the reduction in Ankrd2 impaired the signaling pathways downstream of Akt and MAP kinases. It is well known that these pathways are hyperactivated in many types of cancers: their deregulation contributes indeed to tumor initiation and development, inhibition of apoptosis, angiogenesis, metastasis, and chemoresistance [15,16,17]. We found that Ankrd2 knockdown was responsible for transcriptional and translational downregulation of nuclear lamina components, including lamin A and lamin B, for the alteration of nuclear morphological parameters and for the impairment of DNA integrity. Furthermore, the efficacy of chemotherapeutics was significantly improved upon Ankrd2 reduction, suggesting new therapeutic approaches for the treatment and the cure of cancer cells.

## 2. Results

### 2.1. Ankrd2 Knockdown Impairs Cell Proliferation

**Figure 1:**
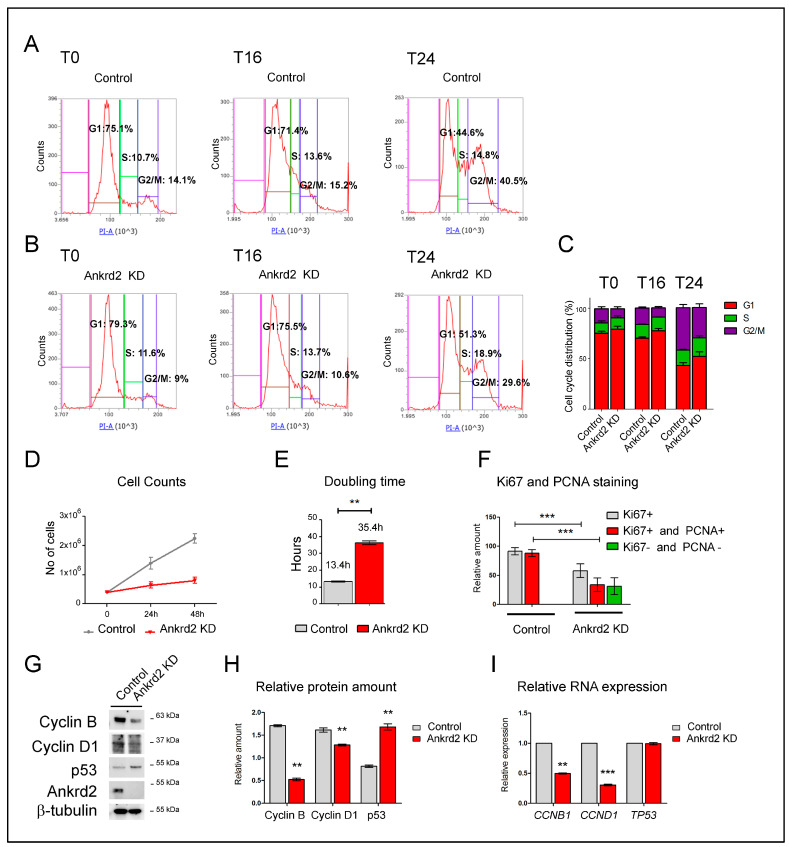
Ankrd2 silencing attenuates cell cycle progression. (**A**,**B**) Representative flow cytometric analysis performed on control (mock-transfected) and Ankrd2-knockdown (Ankrd2 KD) U2OS cells. Cells were synchronized by serum and glucose deprivation and then induced to cycle by mitogenic stimulation for 16 (T16) and 24 (T24) hours. As control, cells were harvested at T0; the percentages of cells in G1, S, and G2/M phases are indicated, and highlighted in the graphs by red, green and purple colors respectively. (**C**) Stacked bar graphs showing the percentage of cells in G1, S, and G2/M phases. Results represent mean values from four independent experiments. Statistical significance of the effects obtained after Ankrd2 silencing was calculated vs. those obtained in control cells. Following the *p* values for Ankrd2 KD vs. control: at T0, G2/M *p* <  0.05; at T16, G1 *p* <  0.05, G2/M *p* <  0.001; at T24, G1 *p*  <  0.05; S *p*  <  0.05; G2/M *p*  <  0.05. (**D**) Cell counts of control (gray line) and Ankrd2-knockdown (Ankrd2 KD, red line) U2OS cells collected at the end of serum deprivation (T0) and after 24 and 48 h of full medium replacement. (**D**) Doubling time (expressed in hours) of cells collected as in (**E**) following Ankrd2 knockdown (** *p* ≤ 0.05). (**F**) Histogram bars relative to Ki67 and PCNA staining of control (mock-transfected) and Ankrd2-knockdown cells. Ki67-positive and Ki67/PCNA-double-positive or -double-negative cells were counted and normalized to the total number of cells of optical fields. Five fields with about 30 cells each were considered. Statistical significance was calculated with an unpaired *t*-test (*** *p* ≤ 0.001). (**G**) Expression of cyclin B, cyclin D1, and p53 in extracts from control and Ankrd2-knockdown (Ankd2 KD) U2OS cells. β-tubulin was used as an equal loading marker. (**H**) Relative densitometric values of cyclin B, cyclin D1, and p53 amounts normalized based on β-tubulin. Statistical significance was calculated with an unpaired *t*-test (** *p* ≤ 0.05). (**I**) Level of *CCNB1*, *CCND1,* and *TP53* transcripts encoding cyclin B1, cyclin D1, and p53, respectively, in control and Ankrd2-knockdown (Ankrd2 KD) U2OS cells. ** *p* ≤ 0.05; *** *p* ≤ 0.001.

Our reported evidence demonstrated that the reduction in the expression of Ankrd2 results in decreased cellular proliferation and clonogenic potential of OS-derived cell lines [14]. This evidence prompted us to investigate the contribution of Ankrd2 to cell cycle progression. To this aim, the level of expression of Ankrd2 was silenced by specific Sh-RNA and Ankrd2-knockdown (KD) clones created accordingly. Ankrd2 KD and control cells were synchronized for 72 h and then induced to proliferate by growth serum supplementation. After 16 and 24 h, cells were collected and processed for flow cytometric analysis. As control, cells were also harvested at T0. Results revealed that, compared to controls, the absence of Ankrd2 resulted in a longer cellular retention in G1, with few cells in S and G2/M phases (Figure 1A,B, T16). Confirming this delay, an increase in cells in G2/M at T24 was reported for control samples only (Figure 1A,B, T24). The impairment of Ankrd2 KD cells for proper duplication was supported by the calculation of their growth rate (Figure 1D) and doubling time (Figure 1E). Specifically, the growth rate was lower in Ankrd2 KD cells, while the doubling time was more than double that of controls. These findings corroborate our earlier results demonstrating that Ankrd2 absence inhibits cell proliferation [14] and provide additional insights into the mechanisms underlying this phenomenon.

Cell proliferation was next evaluated by immunofluorescence, investigating the expression of Ki67 and PCNA. In general, a prevalent Ki67 positivity indicates an active proliferation (in particular from mid-late G1 to early M), while a simultaneous positivity for Ki67 and PCNA identifies cells from late S to late G2. A coincident negativity to Ki67 and PCNA staining indicates cells in non-cycling conditions (G0) or in early G1. Our analysis showed that, compared to the control, Ankrd2 KD cells featured a consistent reduction in Ki67 and Ki67/PCNA staining, and an increased number of cells negative for both markers (Figure 1F). Based on these observations, we reasoned that Ankrd2 silencing may modulate the expression of proteins involved in cell cycle regulation. In particular, we focused on cyclin B and cyclin D1, known for promoting the transition from G2 to M (cyclin B) and from G1 to S phase (cyclin D1). Figure 1G,H demonstrate that Ankrd2 reduction promoted a decrease in cyclin B and D1 levels. At the same time, Ankrd2 silencing also induced an increase in the expression of p53, whose accumulation is generally associated with slowly proliferating cells. RT-qPCR analysis highlighted that the reduction in cyclin B and D1 also occurred at a transcriptional level (Figure 1I). On the contrary, the reduction in the expression of Ankrd2 did not affect the expression of *TP53*, encoding p53 (Figure 1I).

After having established that Ankrd2 silencing impairs cell cycle progression and proliferation, a comprehensive multidisciplinary approach was next exploited to delve deeper into the molecular mechanisms underlying these crucial cellular aspects.

### 2.2. Ankrd2 Reduction Affects the Transduction of Mitogenic Stimulation

**Figure 2:**
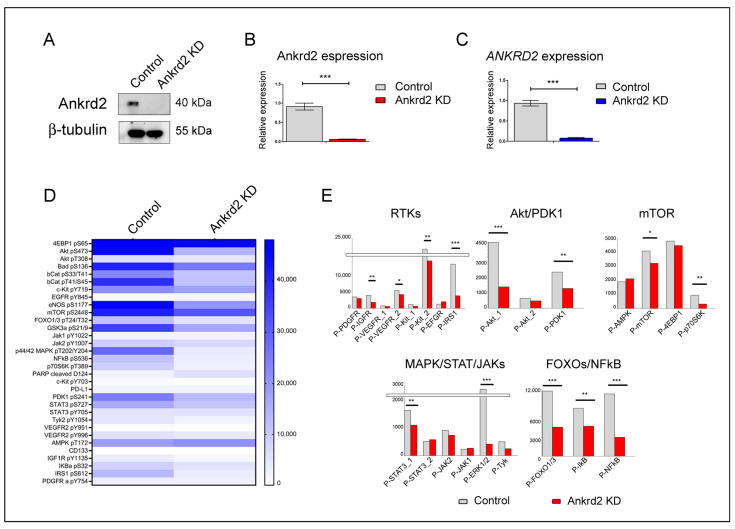
Ankrd2 reduction impairs the response to mitogenic stimuli. (**A**,**B**) Level of expression of Ankrd2 (protein) and (**C**) transcript (*ANKRD2*) in mock-transfected (control) and Ankrd2-knockdown (Ankrd2 KD) cells used for RPPA analysis. *** *p* ≤ 0.001. (**D**) RPPA analysis performed on total lysates from control and Ankrd2-knockdown cells (control and Ankrd2 KD, respectively) stimulated with FCS for 30 min. Values are reported as the mean of signal intensities, with *n* = 3. (**E**) Bar charts displaying optical density values of phosphorylated proteins quantified by RPPA for control (gray) and Ankrd2-knockdown (red) samples, and categorized within five sub-groups of signaling pathways: RTKs, Akt/PDK1, mTOR, MAPK/STAT/JAKs, and FOXOs/NFKB. Mean is only shown. Statistical significance was calculated with an unpaired *t*-test (* *p* ≤ 0.05; ** *p* ≤ 0.01; *** *p* ≤ 0.001). The complete list of antibodies used is reported on Supplementary Table S1.

First of all, we used reverse-phase protein array (RPPA) analysis to investigate Ankrd2’s involvement in signaling cascades relevant to cell cycle progression. Serum-starved control and Ankrd2-knockdown cells were stimulated with mitogens, and RPPA was exploited to simultaneously detect the phosphorylation status of key kinases generally activated with these conditions (Figure 2A–C).

Results showed that, compared to mock-transfected controls, Ankrd2-knockdown cells exhibited a robust decrease in the phosphorylation of proteins belonging to the PI3K/Akt cascade, including PDK1, Akt and its direct effectors GSK3β, Bad, eNOS, and FoxO1 and FoxO3 (Figure 2D,E). Interestingly, a significant reduction in phosphorylation was reported for mTOR and its direct substrate p70S6K (Figure 2D,E). RPPA results also pointed to a significant reduction in the activation of ERK1/2, suggesting an alteration of the signaling cascades upstream of the MAPKs. Another particular feature observed in Ankrd2-knockdown cells was the impaired activation of several Receptor Tyrosin Kinases (RTKs) and downstream adaptors, including IRS1 and Tyk2, which further pointed to the decreased ability of these cells to properly sense and propagate mitogenic stimuli (Figure 2D,E).

### 2.3. Ankrd2 Reduction Impairs Nuclear Morphology and DNA Integrity

**Figure 3:**
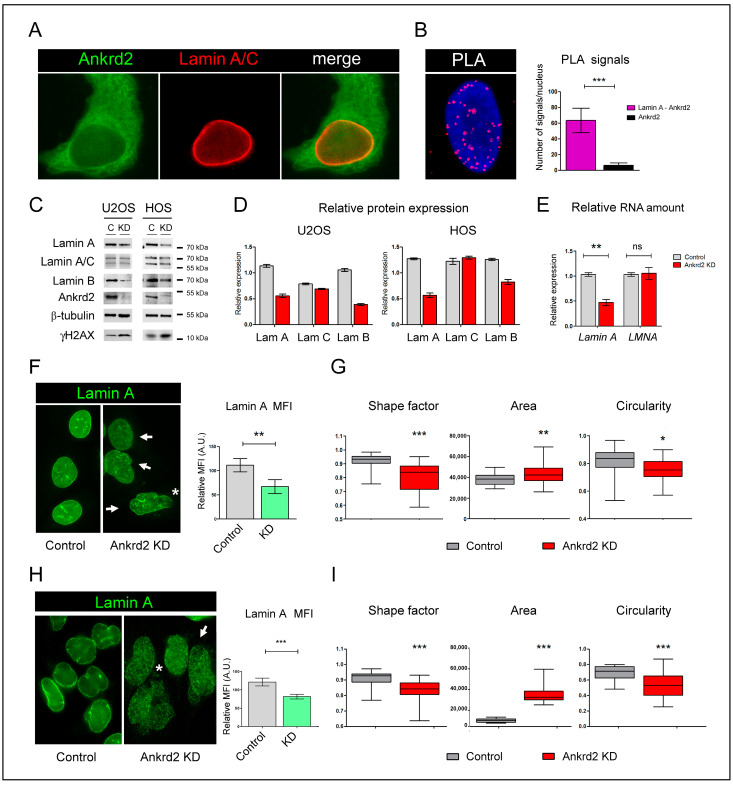
Ankrd2 reduction triggers lamin A reduction and nuclear aberrations in U2OS and HOS cells. (**A**) Immunostaining of Ankrd2 (green) and lamin A/C (red). (**B**) In situ proximity ligation assay (PLA) showing lamin A/C and Ankrd2 interaction (red dots), *** *p* < 0.0001. DNA was counterstained with DAPI. On the right, graphical presentation of PLA signals obtained following incubation with lamin A and Ankrd2 antibodies (purple bar), or as control, with Ankrd2 alone (black bar). (**C**) Western blot analysis showing the level of expression of lamin A, lamin A/C, lamin B, and γH2AX in lysates from control (control) and Ankrd2-knockdown (KD) U2OS and HOS cells. As controls, blots were also incubated with antibodies against Ankrd2 and β-tubulin (loading control). (**D**) Densitometric analysis showing the relative level of expression of lamin A (lam A), lamin C (lam C), and lamin B (lam B) normalized to β-tubulin. (**E**) The real-time PCR analysis of transcripts specific for lamin A or for lamin A and C, which are alternatively spliced products of the same gene (*LMNA*), was performed on RNA isolated from control and Ankrd2-knockdown U2OS cells (control and Ankrd2 KD, respectively). Histograms represent the mean ± SD mRNA fold change (*n* = 3). Statistical analysis was carried out by a one-way ANOVA test. ** *p* < 0.005. (**F**–**H**) Immunofluorescence analysis performed with an antibody specifically raised against lamin A and on control and Ankrd2 KD U2OS (**F**) and HOS (**H**) cells, ** *p* < 0.005. White arrows point to nuclei with alterations in the shape and size; white asterisks highlight nuclear aberrations and ruptures. On the right, the graphical representation of the mean fluorescence intensity (MFI) of lamin A staining in control and Ankrd2-knockdown cells relative to nuclear area, showing a significant decrease in lamin A fluorescence. (**G**–**I**) Morphometric analysis of nuclei of control and Ankrd2 KD U2OS (**G**) and HOS (**I**) cells showing that Ankrd2 reduction perturbs their shape, area, and circularity. Values are reported in pixel. Evaluations were performed by NIS-Element AR software (NIS-Element AR 4.5). Statistical analysis was carried out by two-tailed Student’s t test. *** *p* < 0.0001; ** *p* < 0.008; * *p* < 0.02. (*n* = 100).

Following the demonstration that reduced Ankrd2 expression impairs proliferation and mitogenic response in U2OS cells, another aspect that we deemed particularly relevant for Ankrd2 functions was then investigated. Lamin A and lamin C (lamin A/C) are components of the nuclear lamina. By interacting with several proteins involved in epigenetic modifications, inflammation, and mechanosignaling [18], lamin A/C play a crucial role in many cellular processes such as proliferation, differentiation, migration, and stemness. The importance of lamin A/C is highlighted by the fact that the disruption of their functions is related to the onset of malignancies [19] and genetic disorders, known as laminopathies [20]. Interestingly, previous studies from our laboratory demonstrated that, in muscle cells, lamin A/C interacted with Ankrd2, modulating Ankrd2 shuttling to and out of the nuclei [13]. Therefore, to determine if Ankrd2 also binds to lamin A/C in OS-derived cells, we first conducted an immunofluorescence evaluation on control cells. Co-immunolocalization and proximity ligation assay (PLA) analyses, shown in Figure 3A,B, revealed that lamin A/C and Ankrd2 co-localize at the nuclear rim. To explore the consequences of Ankrd2 reduction on lamin A/C expression and nuclear distribution, Ankrd2 KD cells were then subjected to additional biomolecular investigations. These analyses revealed a selective decrease in lamin A expression (both protein and transcript), as no changes were observed for lamin C (Figure 3C–E). Immunostaining of Ankrd2-silenced cells with an antibody specific for lamin A confirmed the reduction in lamin A expression (Figure 3F), and revealed nuclear abnormalities, such as ruptures, blebs, and alterations in nuclear shape, size, and circularity (Figure 3F,G). Ankrd2 knockdown gave similar effects also in HOS, another human OS-derived cell line (Figure 3C,D,H,I). The destabilizing effect of Ankrd2 silencing on the nuclear lamina also extended to lamin B1, whose expression was significantly reduced in both U2OS and HOS Ankrd2-silenced cells (Figure 3C,D).

Since the loss of nuclear integrity is associated with increased DNA instability [21], we hypothesized that Ankrd2 silencing could also affect DNA integrity. To test this, we examined lysates from control and Ankrd2 KD cells for γH2AX, which increases its amount upon DNA damage. Confirming this hypothesis, results presented in Figure 3C showed that Ankrd2-silenced cells had a higher constitutive level of γH2AX compared to controls.

### 2.4. Ankrd2 Modulates the Cellular Sensitivity to Doxorubicin and Cisplatin

**Figure 4:**
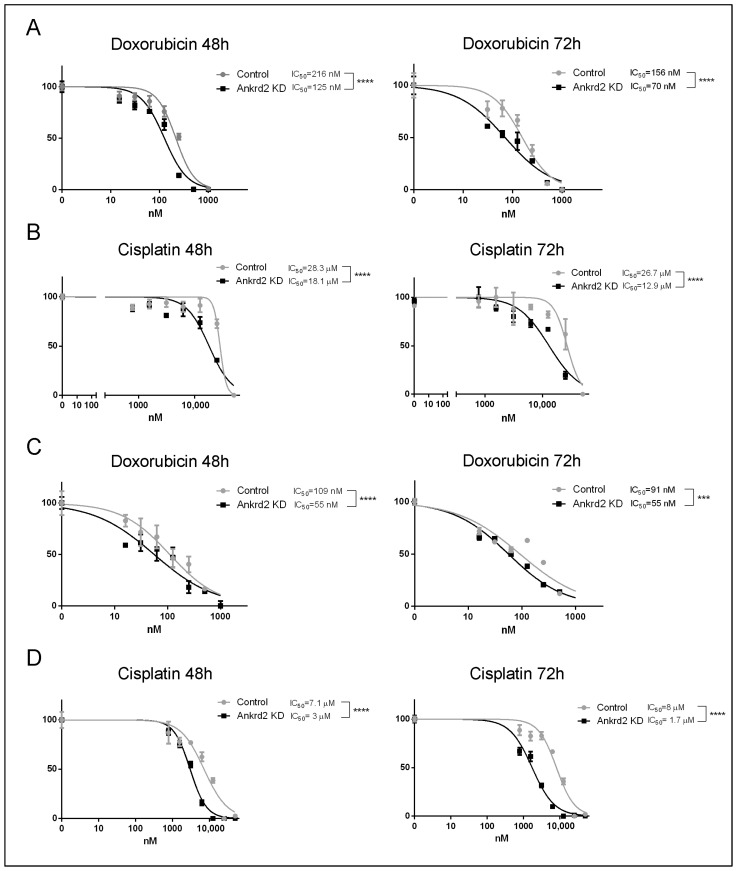
Ankrd2 reduction strengthens the cytotoxic effect of doxorubicin and cisplatin. (**A**,**B**) Viability assays performed on mock-transfected and Ankrd2-knockdown U2OS cells (control and Ankrd2 KD, respectively) treated for 48 and 72 h (48 h and 72 h) with concentrations of doxorubicin (**A**) and cisplatin (**B**) (30–1000 nM and 1–50 µM, respectively). IC_50_ values were calculated by GraphPad Prism 6 software. (**C**,**D**) The same analyses were performed on HOS cells. Three replicates per tested concentration, and at least three independent experiments were performed. Statistical analysis was performed by two-way ANOVA; *** *p* ≤ 0.001, **** *p* < 0.0001.

The reduction in the expression of nuclear lamina components, as well as of cell cycle regulators, along with the loss of DNA integrity that we have observed in Ankrd2-silenced cells, have been linked in the past to increased susceptibility to chemicals that induce replicative stress [22]. In the study of Prof. Pandita’s group, it was indeed reported that lamin A/C-deficient cells, which showed decreased levels of expression of cyclin D1, were particularly sensitive to agents that caused interstrand crosslinks (ICLs) or replication stress [22]. We thus speculated that Ankrd2 reduction might lead to an increased sensitivity to agents that induce DNA damage. To verify this hypothesis, control and Ankrd2-silenced U2OS and HOS cells were subjected to treatment with doxorubicin and cisplatin. These chemotherapeutics, generally used for the treatment of many malignancies including OS [23,24], counteract cellular proliferation by intercalating and crosslinking DNA. The results of the cytotoxicity assays showed that the response to these compounds was improved by Ankrd2 reduction (Figure 4). In particular, calculated IC_50_ values revealed that, in U2OS cells, Ankrd2 silencing reduced doxorubicin sensitivity by half (125 nM vs. 216 nM, Figure 4A) and cisplatin sensitivity by two-thirds (18.1 μM vs. 28.3 μM, Figure 4B). In HOS cells, Ankrd2 silencing improved drugs effects by more than two-fold: for doxorubicin, 55 nM vs. 109 nM (Figure 4C), while 3 μM vs. 7.1 μM for cisplatin (Figure 4D).

## 3. Discussion

Albeit the general interest in exploring the contribution of Ankrd2 to cancer progression is growing [14,25,26,27,28], the definition of its role in cancer cells is still incomplete. Therefore, the main aim of our study has been to investigate the mechanistic role of Ankrd2 in tumor cells’ progression upon Ankrd2 deprivation, using osteosarcoma-derived cell lines.

Consistent with our previous findings [14], our data indicate that reduced Ankrd2 expression impairs cell cycle progression and decreases the cell proliferation index. To investigate the underlying causes of these defects, we found that Ankrd2 silencing led to a downregulation of key cell cycle regulators, specifically cyclin B and cyclin D, which are typically downregulated in non-proliferating cells. Interestingly, their reduction was accompanied by a decrease in the levels of their mRNA. As previously demonstrated, the expression of *CCNB* and *CCND* is promoted by several signaling cascades including those downstream of ERK1/2, Akt, and NF-κB [29]. By demonstrating that Ankrd2 knockdown impairs these signaling pathways, our RPPA results might explain, at least in part, the observed decrease in cyclin B and D expression. On the other side, we cannot rule out that the downregulation of *CCNB* and *CCND* may stem from the activation of p53 following the accumulation of DNA damage seen in Ankrd2-depleted cells (see below). However, given Ankrd2’s reported ability to bind DNA [30], it is also plausible that *CCNB* and *CCND* promoters might be direct targets of Ankrd2 itself. Regardless of how this specific regulation occurs, our observations provide valuable insights into the molecular mechanisms through which Ankrd2 contributes to OS cell proliferation.

Our observations further revealed that OS cells devoid of Ankrd2 showed an increased sensibility to doxorubicin and cisplatin, two of the most common chemotherapeutic drugs used in the treatment of osteosarcoma and other malignancies. In an attempt to understand the biomolecular mechanisms underlying this property, we observed that the reduction in Ankrd2 was related to a cellular increase in the levels of γH2AX, which suggests a persistent basal level of DNA damage.

Our hypothesis is that the cell cycle delay, the lack of a proper mitogenic response, and the increased sensitivity to chemotherapeutics observed in Ankrd2-silenced cells might be a consequence of the nuclear instability driven by the reduction in lamin A and B expression. Previous studies have shown indeed that decreased lamin A and B levels weaken nuclear stability and integrity [22,31], contributing to an increase in DNA damage, possibly through the generation of reactive oxygen species (ROS) [22,32]. This increased DNA damage activates cell cycle checkpoints, halting progression and making cells more vulnerable to additional insults, such as those caused by chemotherapy. However, since recent studies have also demonstrated that persistent DNA damage is also able to induce nuclear envelope rupture through the degradation of lamin A/C [33], the reduction in lamin A/C may be a direct effect of Ankrd2 silencing or a consequence of the increased genome instability that accompanies Ankrd2 depletion.

The nuclear fragility observed in Ankrd2-silenced cells has experimentally precluded to make a clear distinction between Ankrd2-dependent direct and indirect mechanisms for reducing lamin A/C expression. Although we acknowledge the potential involvement of other unexplored pathways, our current efforts focus on developing inducible models to allow for a single and a simultaneous modulation of both Ankrd2 and lamin A.

It is interesting to note that Ankrd2 knockdown was also associated with a specific reduction in lamin A transcript. Since lamin A and lamin C are produced by the alternative splicing of the same gene (*LMNA*), we might speculate that the absence of Ankrd2 might affect the activity of splicing factors, such as serine/arginine-rich splicing factor 2 (SRSF2), whose downregulation leads to a specific decrease in the level of lamin A [34]. Notably, SRSF2 is also activated by Akt [35]. An alternative hypothesis may see Ankrd2 decrease involved in the loss of the stability of the transcript specifically encoding lamin A, possibly through specific microRNAs, including, for example, mir-9 [36]. The involvement of Ankrd2 in modulating lamin A expression is presented here for the first time, and might have a significant impact considering that lamin A plays a crucial role in genetic diseases affecting skeletal muscle [20], in physiological aging [18], and in several types of malignancies [19]. In this regard, the evidence obtained by PLA analysis does not provide detailed information on the nuclear contact points where lamin A and Ankrd2 interact. These could be at the nuclear rim, but also within specific chromatin regions, where Ankrd2 is localized [37]. Further studies will be essential to clarify this aspect, which could provide additional details on the functional role of Ankrd2 in cell and tissue physiology.

Our data also suggest that Ankrd2 depletion could lead to the stabilization of p53 in response to DNA damage. In normal cells, p53 is expressed at low levels in an inactive form [38]; however, in response to DNA stress, p53 is stabilized and accumulates in the nucleus, where it activates genes that halt cell cycle progression, including cyclins D1 and B [39,40], while repressing pro-proliferative genes [41]. Our hypothesis is supported by previously reported data on other models of nuclear instability, in which p53 accumulation resulted from the increased DNA instability and reduced nuclear lamina protein expression [22,42]. Notably, we observed that p53 accumulation in Ankrd2-deficient cells was not accompanied by an increase in *TP53* gene expression, implying that the absence of Ankrd2 may prevent p53 degradation, thereby stabilizing the protein.

Loss of Ankrd2 function could contribute to the increase in sensitivity to chemotherapeutics also through other pathways. For example, NF-κB activation helps cancer cells resist chemotherapy-induced apoptosis [43]. Nonetheless, most chemotherapeutic agents, including doxorubicin and cisplatin, cause reactive oxygen species (ROS) production [44]. Our unreported RPPA results data suggest that Ankrd2 depletion impairs p65 phosphorylation, preventing NF-κB activation and leading to defective ROS scavenging. This may increase vulnerability to apoptosis. Additionally, reduced Akt activity in Ankrd2-silenced cells may impair the Akt-mediated activation of Rad51, a protein crucial for the repair of DNA double-strand breaks induced by doxorubicin and cisplatin [45].

Based on the findings presented here and in our previous study [14], we have hypothesized that reduced Ankrd2 expression may be beneficial for OS patients. To test whether Ankrd2 levels correlate with patient survival, we conducted a Kaplan–Meier analysis on OS patients from the TARGET-OS cohort. However, we found no significant correlation (Supplementary Figure S1). It is important to note that this analysis focused on the canonical Ankrd2 sequence and did not account for potential isoforms, which may limit the interpretation of these results.

Interestingly, data from the TCGA (GDC DataPortal) repository revealed a significant association between low *ANKRD2* expression and improved overall survival in head and neck squamous cell carcinoma (HNSC), lung adenocarcinoma (LUAD), and uveal melanoma (UVM) (Supplementary Figure S2). This suggests that Ankrd2 may have potential as a prognostic biomarker in certain cancers and warrants further investigation.

While the lack of a significant correlation between *ANKRD2* expression and OS patient survival limits the immediate clinical applicability of Ankrd2 as a biomarker or therapeutic target, our study provides a solid foundation for exploring Ankrd2 as a target for combinatorial therapeutic strategies. Further comprehensive studies using patient samples or patient-derived xenograft models will be essential to determine the clinical relevance of Ankrd2 in OS and other cancers.

## 4. Material and Methods

### 4.1. Cell Cultures, Plasmids, and Treatments

Human osteosarcoma-derived U2OS and HOS cell lines were obtained from ATCC-LGC standards Srl, Milan, Italy. Cells were cultured in Iscove’s Modified Dulbecco’s Medium (IMDM)-GlutaMAX (Thermo-Fisher Scientific, Waltham, MA, USA, sold in Italy by Life Technologies, Monza, Italy) supplemented with 10% of heat-inactivated Fetal Bovine Serum (FBS, Thermo-Fisher Scientific). Cells were maintained in a humidified atmosphere with 5% CO_2_ at 37 °C and subcultured twice a week. ANKRD2-silenced stable clones were obtained by transfecting cells with the pSuper.neo + GFP (OligoEngine, Seattle, WA, USA) bearing small double-stranded interfering RNA targeting exons 1 and 2 of the human *ANKRD2* gene already used in the past (14). To exclude any possibility of Sh-RNA off-targets effects, Ankrd2 was also silenced with another interfering RNA targeting exon 4 (5′-TGAAGGTCATTGAGAAGTTCCTGGCTGAC). This Sh-RNA was enclosed in a pRS vector (Origene, Origene Technologies GmbH, Herford, Germany, sold in Italy by TEMA Ricerca, Castenaso, Italy). MTT assays performed on Ankrd2-KD U2OS clones obtained with the latter Sh-RNA showed the same sensibility to doxorubicin and cisplatin observed with Sh-RNA targeting exons 1 and 2, thus confirming the reliability of the results obtained with the first vestor (Supplementary Figure S3). Transfected cells were isolated, and single-cell clones expanded in the presence of G418 (Life Technologies) as described in [14]. The obtained stable clones were screened by PCR and WB analyses. For RPPA and cell cycle analysis, cells were synchronized for 72 h by serum and glucose deprivation and then induced to proliferate in normal growth medium for the indicated times. Doxorubicin and cisplatin were purchased from Merck (Merck-Sigma, St. Louis, MO, USA) and used as indicated in the text.

### 4.2. Cell Cycle Analysis

Parental, control (mock-transfected), and Ankrd2-knockdown cell lines were maintained in low-glucose and serum-free medium (Thermo-Fisher Scientific) for 3 days and then released by serum addition for the times indicated. Using this synchronization protocol, the G1-phase compartment, which generally constitutes 40% to 45% of the total population, was considerably enriched, reaching 80%. At the times indicated, cells were harvested by trypsinization, washed, fixed with ethanol 70%, and incubated with 0.5 μg/mL Propidium Iodide (Merck Life Science S.r.l., Milan, Italy). Cell cycle distribution was evaluated in 10,000 cells, using an Attune Nxt Acoustic Focusing Cytometer (Thermo-Fisher Scientific) equipped with a blue laser (488 nm). Data were acquired in list mode using Attune Cytometric 2.6 software (Thermo-Fisher Scientific).

Doubling time was calculated by the application of the formula described by Vidal and colleagues [46]:

Doubling time = [T × (ln^2^)]/[ln (**Xe**/**Xb**)]

where T = time in any units, Xe = number of cells after T (days or hours), and Xb = number of cells at T0.

### 4.3. Protein Extracts and Immunoblot

Cells were lysed in SDS lysis buffer [14]. Total lysates were resolved by SDS-PAGE, electro-transferred onto nitrocellulose membranes (Santa Cruz Biotechnology, DBA Italia SRL, Segrate, Italy), and immunoblotted with the following antibodies: lamin A (ab26300 polyclonal, Abcam Cambridge, UK), lamin B (clone 8F10.1, Merck-Sigma, St. Louis, MO, USA), p53 (clone Ab-6 Merck-Sigma), β-tubulin and pan-actin (both from Merck-Sigma), Ankrd2 (polyclonal, Proteintech, DBA, Milan, IT); γH2AX and P-Akt Ser473 (Cell Signaling Technologies, Danver MA, USA); Akt, and cyclin D1 (Santa Cruz Technologies, DBA Milan, IT). HRP-conjugated secondary antibodies were from Invitrogen (Thermo-Fisher Scientific, Waltham, MA, USA).

### 4.4. Quantitative Real-Time PCR

Total RNA was extracted and purified using the PureLink™ RNA Micro Kit (Invitrogen, Life Technologies, Monza, Italy) according to manufacturer’s instructions. RNA integrity and quantification were analyzed and 1 μg of total RNA was next reverse-transcribed to cDNA using the High-Capacity cDNA Reverse Transcription Kits (Applied-Biosystems, Thermo Fisher Scientific, Waltham, MA, USA). Quantitative real-time PCRs were performed using QuantStudioTM 3 Real-Time PCR System (Applied Biosystems, Thermo Fisher Scientific, Waltham, MA, USA) and the PowerTrack™ SYBR™ Green Master Mix (Applied Biosystems, Thermo Fisher Scientific, Waltham, MA, USA) by using the following oligonucleotides:

human *RPLP0*
(F: TACACCTTC CCACTTGCTGA, R: CCATATCCTCGTCCGACTCC);
human *ANKRD2*
(F: CGGTTATGGACGGCACCAT, R: CTTCTCATCCTCCAGCACCA);
human *Lamin A*
(F: CTCCTACCTCCTGGGCAACT, R: AGGTCCCAGATTACATGATGCT);
human *LMNA*
(F: GCAAAGTGCGTGAGGAGTTT, R: GAGTTCAGCAGAGCCTCCAG);
human *CCND1*
(F: CATCTACACCGACAACTCCATC, R: TCTGGCATTTTGGAGAGGAAG);
human *CCNB1*
(F: CCTCCCTTTTCAGTCCGC, R: CTCCTGTGTCAATATTCTCCAAATC).

Relative gene quantification was performed using the comparative threshold (Ct) method (ΔΔCt), where the relative gene expression level equals 2^−ΔΔCt^. The obtained fold changes in gene expression were normalized to the housekeeping gene hRPLP0. Experiments were performed in triplicate, and the results expressed as means ± standard deviation. Differences among three or more groups were analyzed through an *ANOVA, followed by a Newman–Keuls post hoc test with *p* < 0.05 considered as statistically significant.

### 4.5. Immunofluorescence

Cells were seeded on glass coverslips and treated according to the experimental protocols. At the end of each treatment, cells were fixed in methanol and unspecific signal reduced by incubation with 5% Bovine Serum Albumin (BSA, Merck Life Science S.r.l., Milan, Italy) in PBS. Glass-coverslips were incubated with primary antibodies and then with the related secondary antibody conjugated with FITC or TRITC fluorochromes. Antibodies used were mouse anti-lamin A/C (clone E1, from Santa Cruz Biotechnology, DBA Segrate, Italy) and anti-Ankrd2 (Proteintech, DBA Segrate Italy). In situ proximity ligation assay (PLA) was performed using Duolink^®^ In Situ Detection Reagents Orange (DUO92007) (Merck-Sigma, Merk Life Science S.r.l. Milan, IT) as previously described [47]. Quantitative analysis of PLA results was performed using Duolink Image Tool software version 1.0.1.2 (Merck-Sigma) by counting 50 nuclei. Samples were observed with a Nikon Eclipse epifluorescence microscope. Morphometric analyses, shape factor, and circularity were calculated by NIS-element AR software, applying the following formula: shape factor = (perimeter)^2^/area and circularity = 4π × area/(perimeter)^2^.

### 4.6. Reverse-Phase Protein Array (RPPA)

RPPA is a high-throughput antibody-based targeted proteomic platform that can simultaneously quantify proteins and their post-translational modifications, such as phosphorylation, as a measure of their activity, providing a snapshot of cellular protein activity, upon a particular treatment or condition. Protein samples are arrayed as microspots on nitrocellulose-coated glass slides. Each slide is probed with a specific antibody that can detect levels of total protein expression or post-translational modifications [48]. In our experiments, after treatments, cells were lysed in RIPA-like lysis buffer (LB) composed of tissue protein extraction reagent (T-PER, Thermo-Fisher Scientific, Waltham, MA, USA) supplemented with 300 mM NaCl and protease and phosphatase inhibitor cocktails (Merck Millipore, Burlington, MA, USA). Total protein content was next quantified. RPPA samples were prepared as previously described [49]. The complete list of the antibodies enrolled in this analysis is reported in Supplementary Table S1.

### 4.7. MTT Assay

To test the effects of chemotherapeutic agents, U2OS and HOS cell lines were cultured for 48 and 72 h in the presence of the vehicle (DMSO 0.1%) or increasing concentrations of doxorubicin and cisplatin (0–1 μM and 0–50 μM, respectively). Cell proliferation was determined using the MTT cell proliferation kit (Roche Diagnostic, Basel, Switzerland), following the manufacturer’s instructions. Briefly, 0.5 mg/mL of MTT labeling reagent was added to each well and incubated for 4 h. Purple formazan crystals were solubilized by adding 100 μL of the solubilization solution (0.01 M HCl and 10% SDS) overnight. The plate was subsequently read on an Infinite M200 photometer (Tecan Group Ltd., Mannedorf, Switzerland) at a wavelength of 570 nm. Colorimetric readings were normalized against plates of untreated cells under identical culture conditions.

### 4.8. Analysis of Clinical Data

Overall survival curves related to *ANKRD2* expression were performed by BMR Genomics, Padua Italy (https://www.bmr-genomics.it/), accessed on 29 March 2024. Osteosarcoma analyses were conducted on TARGET-OS database (version 40.0, 29 March 2024). For this analysis, *ANKRD2* expression values were plotted for metastatic (*n* = 22) and primary (*n* = 65) tumor samples. *ANKRD2* expression values were also plotted for categories of patients’ age at the onset of the diagnosis, that is, pediatric (0–14 years old (y.o.), *n* = 23), teen (15–18 y.o., *n* = 32), young adults (15–39 y.o., *n* = 15), and adults (>30 y.o., *n* = 3). Curves are shown in Supplementary Figures S1 and S2).

### 4.9. Image Processing and Statistical Analysis

Morphological and biochemical images were processed by Photoshop CS4 (Adobe Systems). Densitometric analyses were performed by ImageJ version 1.52a (National Institute of Health), while morphometric evaluations by NIS-element software, version AR 4.5. Except where indicated, data are expressed as the mean ± SD of the number of the reported biological replicates. Statistical significance was measured by Student’s *t* test (comparison of two groups). Statistical analyses were carried with GraphPad Prism version 5.0 for Windows (GraphPad Software). Results were considered statistically significant for values less than 0.05.

## Data Availability

Data are available upon request to the corresponding author.

## Supplementary Materials

The following supporting information can be downloaded at: https://www.mdpi.com/article/10.3390/ijms26041736/s1.
