# Supplementary material for: ANKRD2 Knockdown as a Therapeutic Strategy in Osteosarcoma: Effects on Proliferation and Drug Response in U2OS and HOS Cells
Source: Int J Mol Sci. 2025 Feb 18;26(4):1736. doi: 10.3390/ijms26041736 (PMC11855734; doi:10.3390/ijms26041736)
Supplement: Supplementary file 1 [file ijms-26-01736-s001.zip › ijms-3476955-supplementary.pdf]

Supplementary Material for:

ANKRD2 Knockdown as a Therapeutic Strategy in Osteosarcoma: Effects on Proliferation and Drug Response in U2OS and HOS Cells

Vittoria Cenni et al.

Table S1

Complete list of antibodies used for RPPA analysis.

All antibodies were from cell signaling technologies

1. 4EBP1 pS-65
2. Akt pS-473
3. Akt pT-308
4. Bad pS-136
5. bCat pS-33/pT-41
6. bCat pT-41/pS-45
7. c-Kit pY-719
8. EGFR pY-845
9. eNOS pS-1177
10. mTOR pS-2448
11. FOXO1/3 pT-24/pT-32
12. GSK3a/b pS-21/pS-9
13. Jak1 pY-1022
14. Jak2 pY-1007
15. p42/44MAPK pT-202/pY-204
16. NFkB pS-536
17. p70S6K pT-389
18. PARP cl D124
19. c-Kit pY-703
20. PD-L1
21. PDK1 pS-241
22. STAT3 pS-727
23. STAT3 pY-705
24. Tyk2 pY-1054
25. VEGFR2 pY-951
26. VEGFR2 pY-996
27. AMPK pT-172
28. CD133
29. IGF1R pY-1135
30. IKBa pS-32
31. IRS1 pS-612
32. PDGFRa pY-754

Figure S1

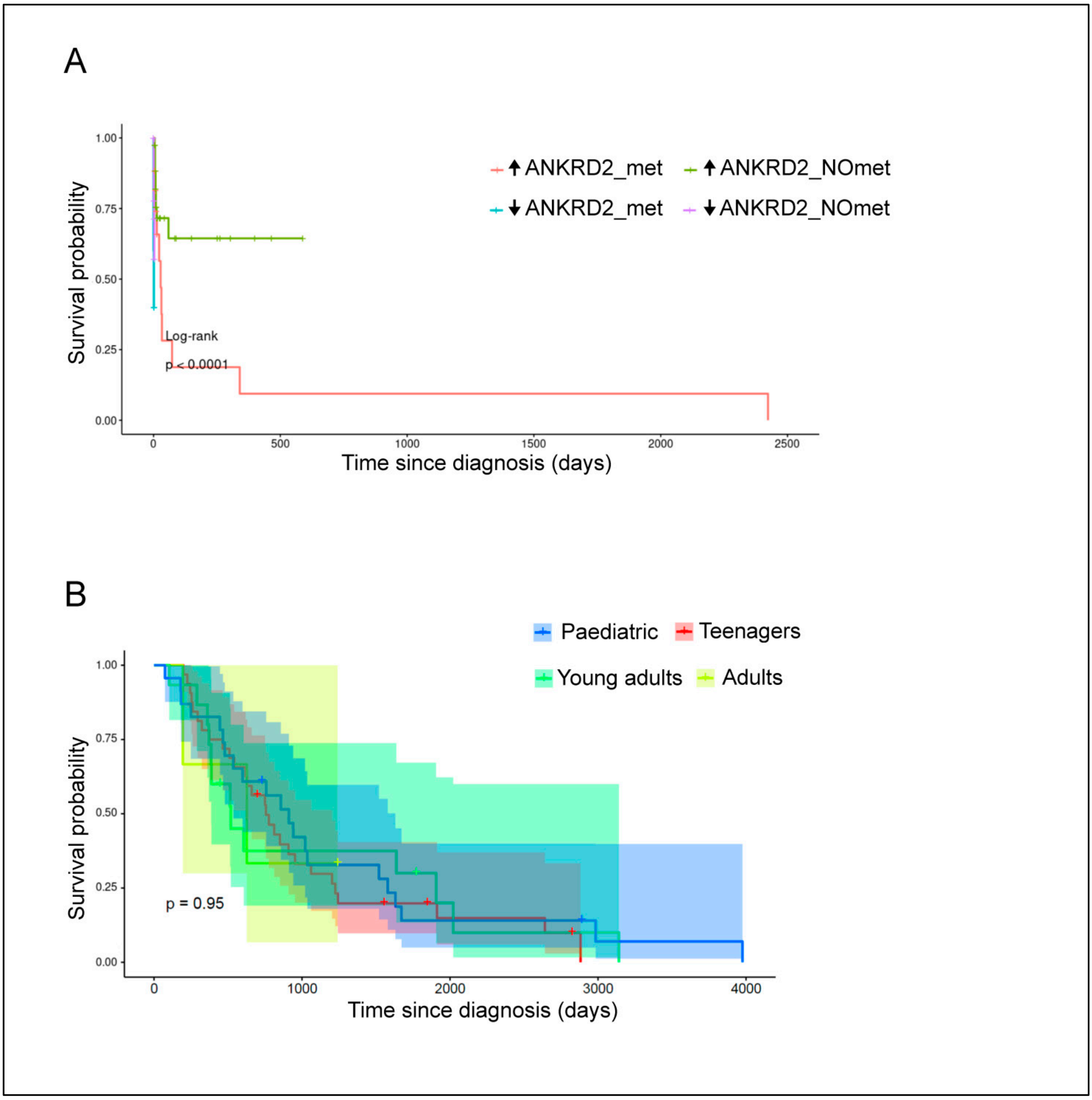

Figure S1: *ANKRD2* expression does not correlate with the overall survival of patients affected by osteosarcoma. **A)** Kaplan-Meier curve generated with data from TARGET-OS database to determine the association of *ANKRD2* expression with the overall survival of osteosarcoma patients. Stratification was performed for patients expressing high or low amount of *ANKRD2* (↑*ANKRD2* and ↓*ANKRD2* respectively) and for the presence or absence of metastasis (met and NOmet, respectively). Significance of these curves indicates that the analysis has been properly performed but *ANKRD2* expression does not predict survival. **B)** Kaplan-Meier curve generated with data from TARGET-OS database of patients affected by primary osteosarcoma. Patients were grouped in four aged-matched classes: paediatric (age 0-14), teen age (15-18), young adult (15-39) and adult (>39).

Figure S2

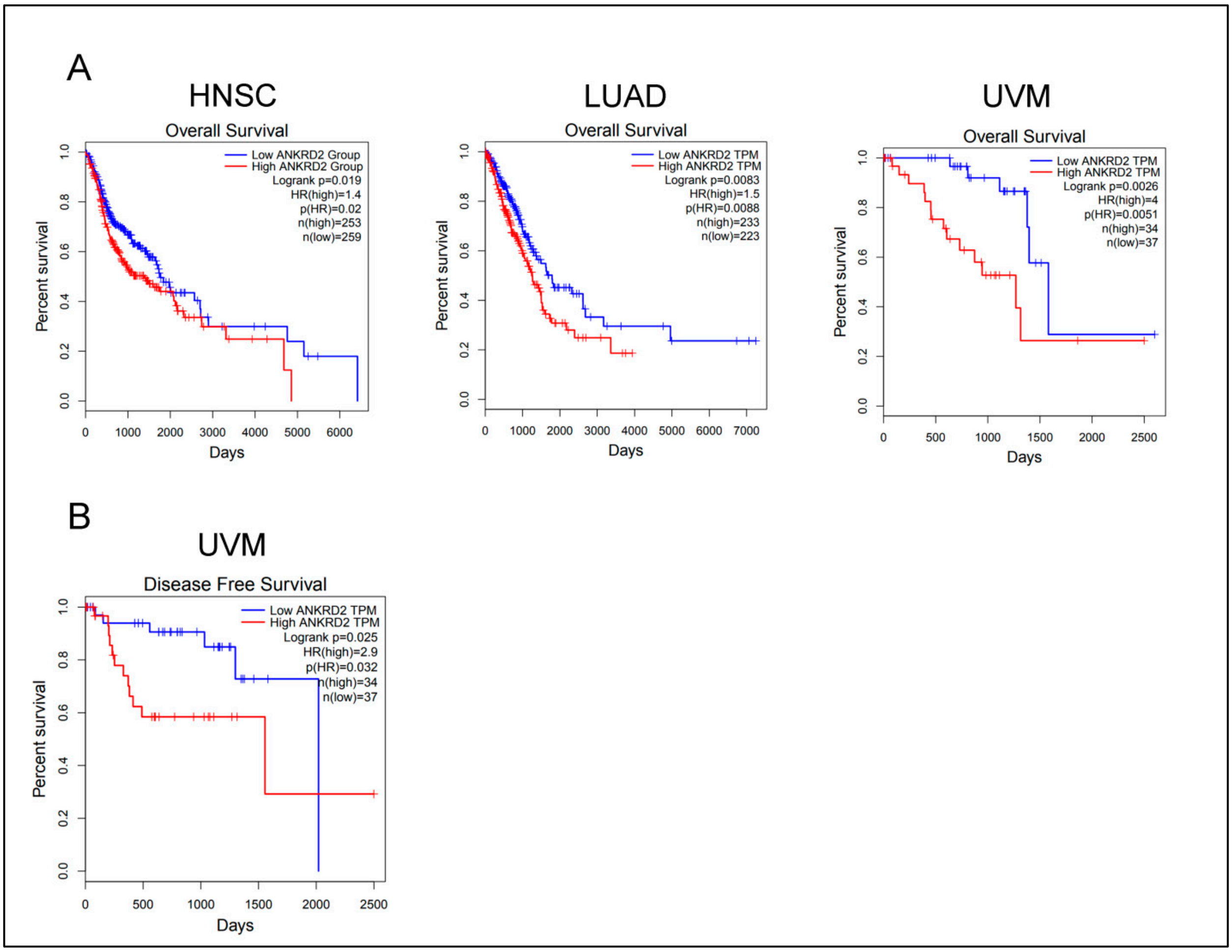

Figure S2: *ANKRD2* levels predict overall and disease-free survival in a selected group of malignancies. A) Kaplan-Meier curves showing overall survival in head and neck squamous cell carcinoma (HNSC), lung adenocarcinoma (LUAD) and uveal melanoma (UVM) in correlation with *ANKRD2* expression. **B)** Disease free survival plots of UVM relative to *ANKRD2* expression.

Figure S3

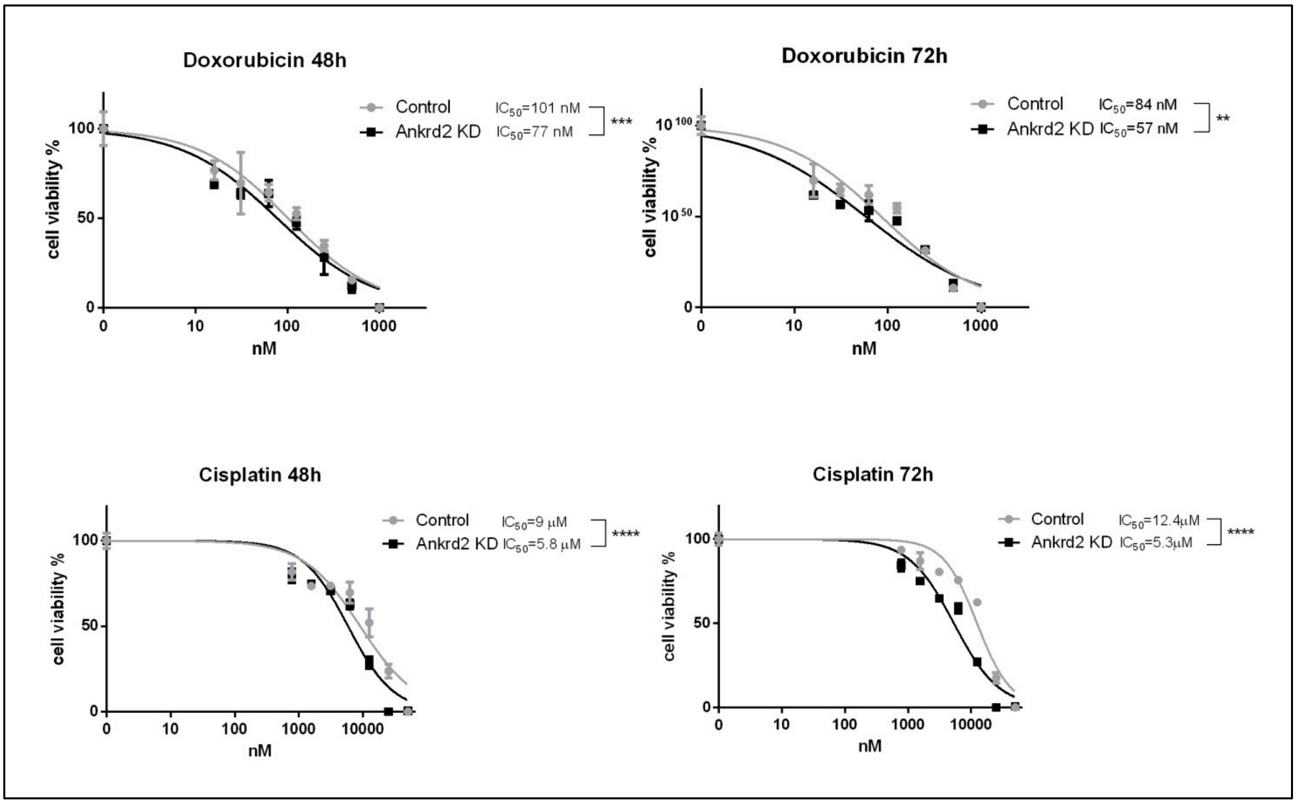

Figure S3: Response to doxorubicin and cisplatin of U2OS cells in which *ANKRD2* was silenced with a Sh-RNA targenting exon 4 of the human sequence of *ANKRD2*. Viability assay performed on U2OS clones transfected with mock-pRS vector (control) and pRS-Sh-ANKRD2 (Ankrd2 KD), targeting exon 4 of the human ANKRD2 sequence (from Origene technologies): Cells were treated for 48 and 72 hours (48h and 72h) with concentrations of doxorubicin and cisplatin (30-1000nM and 1-50μM respectively). IC_{50} values were calculated by GraphPad Prism 6 software. Three replicates per tested concentration and two independent experiments were performed. Statistical analysis was performed by two-way Anova; **** $p < 0.0001$.

Original Microscopy and blots used within the paper. Dimension, brightness and luminosity of the images were elaborated with Adobe Photoshop 7.0.

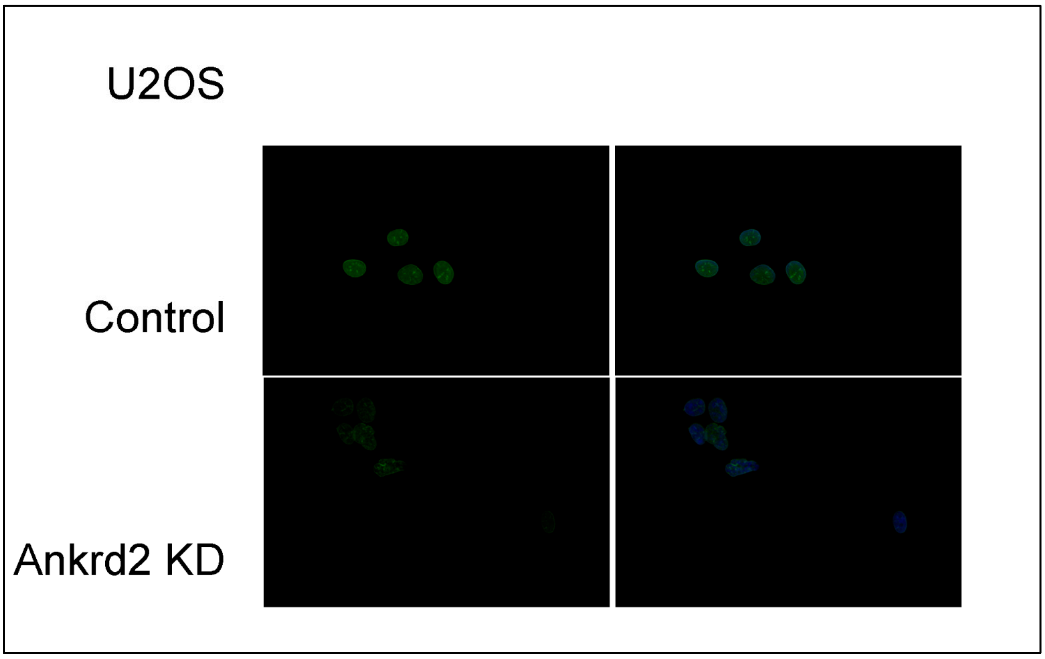

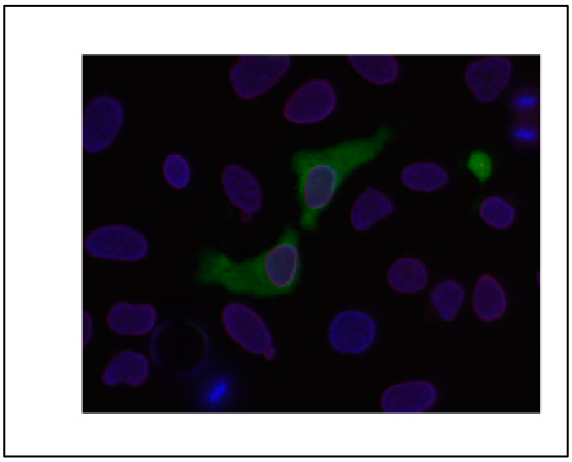

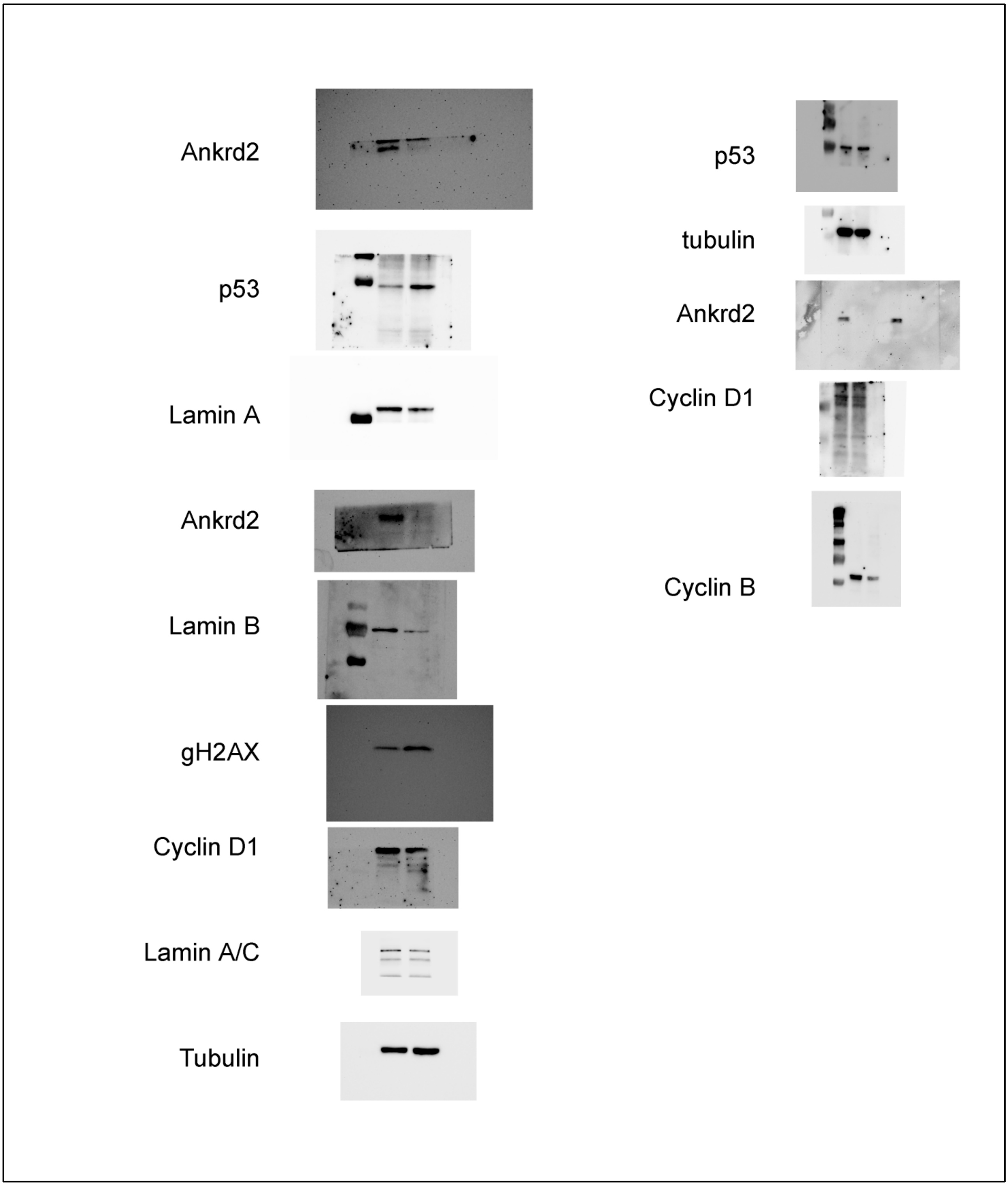
Ankrd2
p53
Lamin A
Ankrd2
Lamin B
gH2AX
Cyclin D1
Lamin A/C
Tubulin
p53
tubulin
Ankrd2
Cyclin D1
Cyclin B

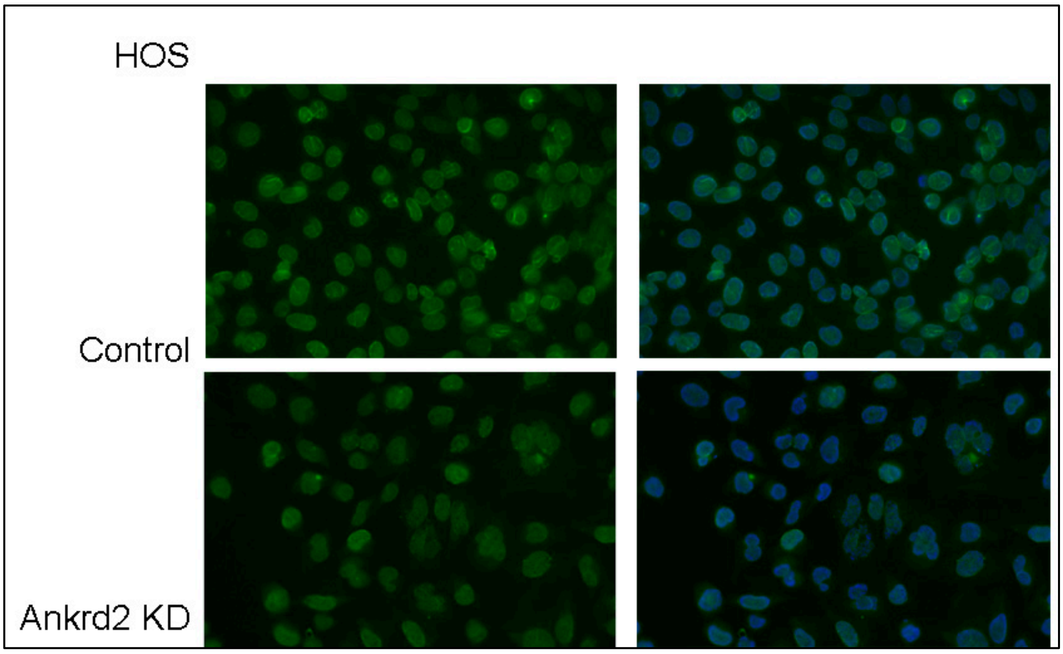
HOS
Control
Ankrd2 KD

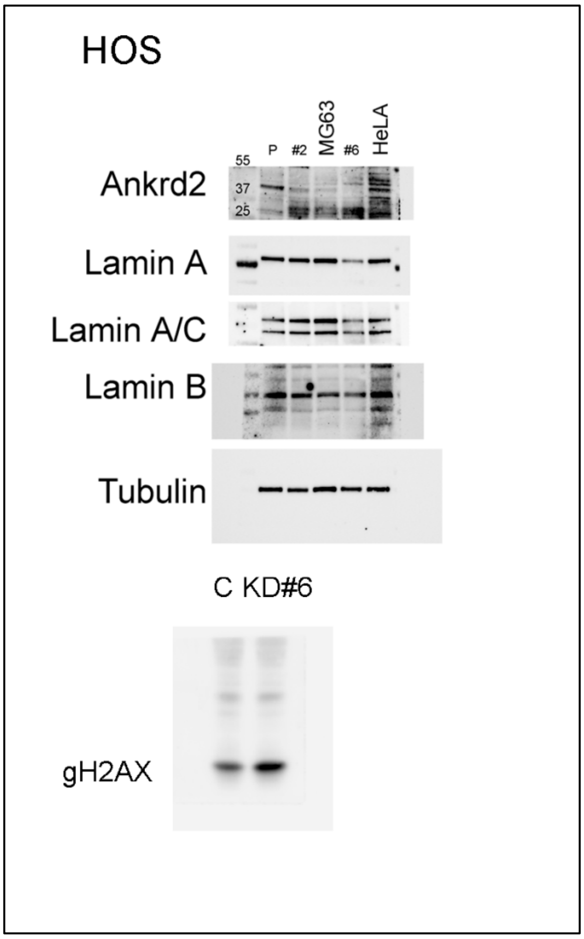
HOS
55
37
25
P
#2
MG63
#6
HeLA
Ankrd2
Lamin A
Lamin A/C
Lamin B
Tubulin
C KD#6
gH2AX
